# A 12-month follow-up of a transdiagnostic indicated prevention of internalizing symptoms in school-aged children: the results from the EMOTION study

**DOI:** 10.1186/s13034-020-00322-w

**Published:** 2020-04-22

**Authors:** M. E. S. Loevaas, S. Lydersen, A. M. Sund, S-P. Neumer, K. D. Martinsen, S. Holen, J. Patras, F. Adolfsen, L-M. P. Rasmussen, T. Reinfjell

**Affiliations:** 1 Department of Psychology, NTNU, Norwegian University of Science and Technology, Trondheim, Norway; 2 Department of Child and Adolescent Psychiatry, St. Olavs University Hospital, Trondheim, Norway; 3 Medical Faculty, Department of Mental Health, Regional Center for Child and Youth Mental Health and Child Welfare, NTNU Norwegian University of Science and Technology, Trondheim, Norway; 4 Centre for Child and Adolescent Mental Health, RBUP East and South, Oslo, Norway; 5 Regional Centre for Child and Youth Mental Health and Child Welfare, UiT Arctic University of Norway, Tromsø, Norway

**Keywords:** Anxiety, Depression, Follow-up, Transdiagnostic prevention, Child

## Abstract

**Background:**

Anxious and depressive symptoms in youth are highly prevalent, are often comorbid and have a high rate of relapse. Preventive interventions are promising, but follow-up results are lacking. The transdiagnostic EMOTION program is an indicated preventive cognitive behavioral therapy (CBT) intervention targeting children aged 8–12 years.

**Methods:**

The present study investigates the 12 months follow-up effects of the EMOTION intervention in a cluster randomized controlled trial (RCT) with 795 children that included both child self-reports and parental reports.

**Results:**

Mixed model analyses showed a larger decrease of symptoms in the intervention group than in the control group for child self-reported anxious symptoms (The Multidimensional Anxiety Scale for Children (MASC) difference 4.56, CI 1.83 to 7.29, *p *= .001). Parental reports for both anxious (MASC difference 2.50, CI .26 to 4.74, *p *= .029) and depressive (The Mood and Feelings Questionnaire-short form (SMFQ) difference 1.55, CI .83 to 2.26, *p *≤ .001) symptoms in children also showed a reduction. No statistically significant difference was found for child self-reported depressive symptoms (SMFQ difference .69, CI − .22 to 1.60, *p *= .139).

**Conclusion:**

The transdiagnostic EMOTION program has shown the potential for long-term reductions in symptoms of both anxiety and depression in school-aged children. However, results regarding depressive symptoms must be considered preliminary as only parental report indicated effect.

*Trial registration* The regional ethics committee (REC) of Norway approved the study. Registration number: 2013/1909; Project title: Coping Kids: a randomized controlled study of a new indicated preventive intervention for children with symptoms of anxiety and depression. ClinicalTrials.gov Identifier; NCT02340637.

## Background

Anxious and depressive disorders (i.e., internalizing disorders) are common and often comorbid in youth [1]. Research indicate that comorbid anxious and depressive symptoms are associated with an increased risk of developing anxiety and depressive disorders in the future [2], and relapse rates for these disorders are high [3, 4]. Internalizing disorders in youth are often associated with functional impairments in important life areas, such as academic achievement and social functioning, and even subclinical symptoms have been found to influence adaptive development negatively [5–7]. Internalizing disorders tend to be stable in children over time [1, 2]. Hence, it is necessary to identify interventions that can interrupt this process, with the aim of reducing or minimizing possible negative consequences associated with these disorders. Successful prevention has the potential to reduce the severity and persistence of symptoms for those affected and to decrease the incidence of new diagnosable cases [3].

The majority of prevention research targeting children with internalizing symptoms focus on programs based on cognitive behavioral therapy (CBT) that are delivered in a school setting [3, 8]. A recent review [3] reported positive results after indicated interventions, that is, interventions aimed at youth with elevated symptoms. These interventions were successful at reducing symptoms of both anxiety and depression in youth; however, follow-up periods of up to 12 months revealed limited long-term effects. The authors identified important areas for improvement, especially the need for follow-up studies [3]. Similarly, Werner-Seidler et al. [4] reviewed studies of both anxiety and depression and found small positive effects for school-based prevention programs for youth both at postintervention and at follow-up. Indicated programs were found to be more effective for depression than universal programs. No difference between the indicated and universal programs was detected for anxiety. The same was true for follow-up effects, however the small number of follow-up studies measuring anxiety symptoms reduces the reliability of the results reported in this review [4]. In another meta-analysis of anxiety prevention programs [9], the results indicated small effect sizes post intervention and at 6 months follow-up; at the 12-month follow-up, however, the effect had diminished. Conflicting results were recently published by Rasing et al. [10], who found no postintervention effect for indicated CBT-based anxiety prevention programs delivered in groups. They did find a significant decrease in anxious symptoms at 6 months postintervention. This effect diminished after 12 months, however, similar to the results reported by Fisak et al. [9].

Several studies focusing on depression have found positive short- and long-term effects for indicated prevention programs [3, 11]; however, the effects seem to diminish over time. Similarly, in a recent meta-analytic review with at-risk adolescents, no effects were found for depression at follow-up after 6 and 12 months [10]. The effect of preventive interventions targeting both anxious and depressive symptoms was not moderated by the participants’ age or gender [4, 9, 12].

In summary, the effects of indicated preventions are generally promising, although heterogeneity is high. Follow-up results are scarce, especially regarding prevention of anxiety, indicating that more studies are needed. Implementation of the intervention is also considered to be an important part of successful prevention. Still, more research into feasibility and readiness for implementation are important and could contribute to improved effectiveness of prevention interventions in the school-system, something that has been the focus of recent research [13, 14].

Transdiagnostic interventions have the advantage of targeting both anxious and depressive symptoms concurrently, thus reaching a broader population and simplifying implementation for professionals as only one program has to be learned and implemented [15, 16]. Transdiagnostic approaches are developed based on the common comorbidity of these disorders and the similarities between the disorders in terms of etiology, risk factors, and treatment strategies [15, 17]. Crossover effects from prevention programs that focus exclusively on either anxiety or depression further support the idea of targeting both symptom categories with the same intervention [18]. There are few studies of such interventions for youth, but the results to date are generally promising [19–22]. In addition, there are studies that focus on the prevention of both anxiety and depression without using the term “transdiagnostic”; one example is the Aussie Optimism; Positive Thinking Skills Program for the prevention of depression and anxiety in school-aged children. This program reported positive short-term results for depression, but not anxiety, when implemented in schools with youth defined as high risk. No effects were identified at a 30-month follow-up [23, 24]. The comparable program FRIENDS for Life is also aimed at preventing anxiety and depression and has shown positive effects after 6 and 12 months for both anxiety and depressive symptoms when used as an indicated program [25]. Although evidence so far is scarce, the results indicate the potential for positive long-term effects after implementing a transdiagnostic preventive intervention.

The present study examined the long-term effect of the newly developed transdiagnostic indicated prevention program EMOTION “*Coping Kids*” *Managing Anxiety and Depression* [26, 27] in Norwegian school children aged 8 to 12 years with high self-reported levels of anxious and/or depressive symptoms. EMOTION is a CBT-based program aiming to reduce anxious and depressive symptoms in school-aged children. The postintervention effects of the EMOTION program have shown positive results for child self-reports of anxious and depressive symptoms as well as parent-reported depressive symptoms in children. Parental reports of child anxiety symptoms, however, did not show any significant difference between the groups from pre- to postintervention [28].

Based on the positive postintervention results of EMOTION [28], we hypothesized that the intervention group would decrease significantly more than the control groups in child self-reported anxious and depressive symptoms at the 12-month follow-up compared to baseline. In addition, we hypothesized that parental reports of their child’s depressive symptoms would reflect a decrease in symptoms and possibly also a decrease in parent-reported anxious symptoms. We further investigated whether the differences between the groups continued to increase from postintervention to the 12-month follow-up for both child and parental reported symptoms.

## Method

### Procedure

The data in the present study are part of the Coping Kids evaluation in Norway, a cluster randomized controlled study (RCT) of a new, indicated preventive intervention for children, the EMOTION program [26, 27]. The data sample was collected from 36 public schools, covering both rural and urban areas in Norway. A representative from the Coping Kids project group presented the study to school leaders, who further volunteered their school for participation. Schools were not given any funding to participate in the study.

**Fig. 1:**
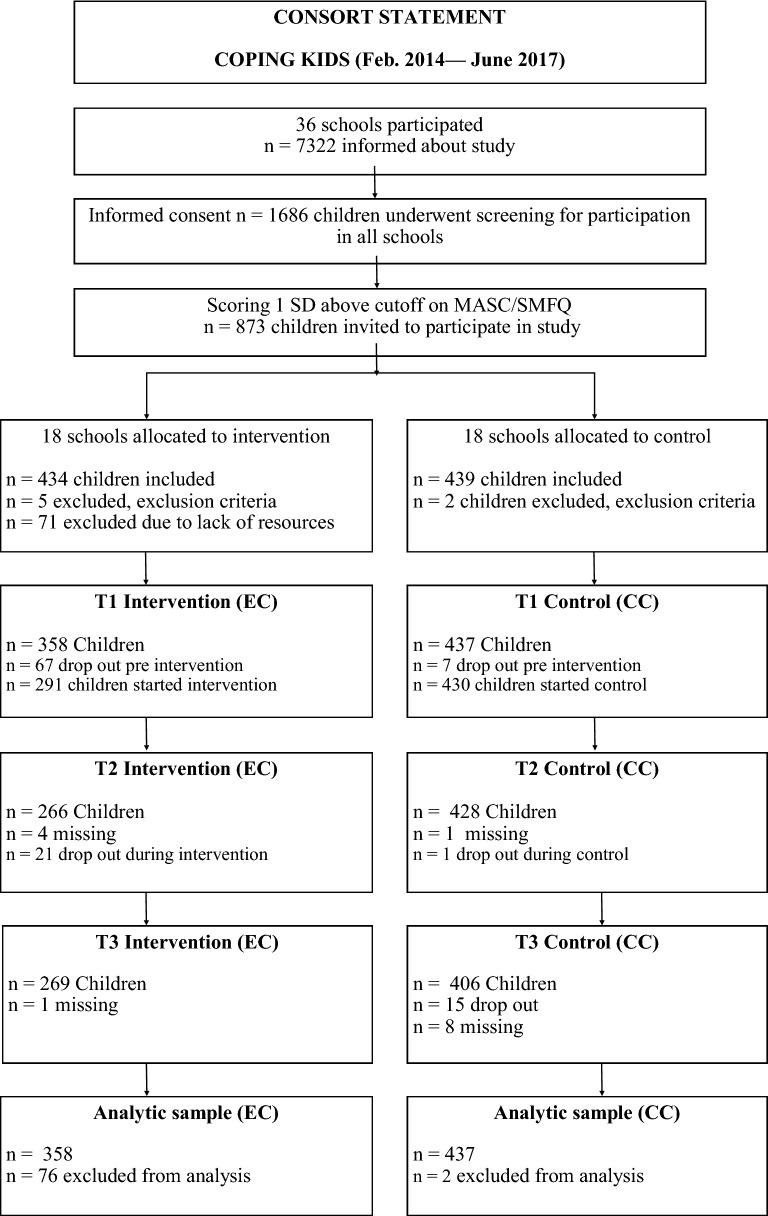
A flowchart of participants through the study. In the intervention condition; four children were sick at T2, but present at T3

Prior to randomization the schools were matched on geographic location, size and demographic factors. Schools were then randomized into 18 intervention schools and 18 control schools. The data were collected between fall 2014 and spring 2017, with enrollment of new children occurring every semester. For each child, data were collected at three waves: before the intervention, immediately after the intervention was completed (approximately 10 weeks) and at follow-up 1 year after the intervention was completed (see Fig. 1, Consort Statement). All data were collected electronically. Power calculations were done prior to the main study, and the required sample was 559 children [29].

An invitation to participate in screening was handed out to all children between the ages of 8 to 12 years (corresponding to grades 3, 4, 5 and 6) at the participating schools. Participation in screening required expressed interest from the child and a signed consent form from the caregivers. The children answered the questionnaires digitally at the schools, and teachers were available to answer questions. Children who scored above a predetermined cut-off on either anxious (The Multidimensional Anxiety Scale for Children, child version (MASC-C) ≥ 61 points for girls and ≥ 54 points for boys [30]) or depressive symptoms (The Mood and Feelings Questionnaire—short form, child version (SMFQ-C) ≥ 7 points regardless of gender [31]) were invited to participate in the study. The parents of the included children were invited to participate, and link to questionnaires were sent to consenting parents by email.

Group leaders, with different professional background (e.g. health nurses, educational and psychological counselors and psychologists) were recruited from primary or secondary health services. Two group leaders delivered the EMOTION program to groups of three to seven children two times a week for 10 weeks. Child groups were held at the schools, during school hours or immediately after. In addition, parent groups were held seven times over the 10-week period, with children participating in four of the meetings. All parental meeting was held in the afternoon at the school premises. The group leaders underwent a three-day training seminar that covered basic CBT and the EMOTION manual. A trained CBT supervisor provided 10 h of supervision to the group leaders over the intervention period.

To ensure fidelity to the program 17% of the sessions were videotaped and rated (from 0 = None to 6 = Thorough) using the Competence and Adherence for Cognitive Behavioral Therapy (CAS-CBT) [32] and fidelity were supported (M = 3.55, SD = 1.24) [33]. Group leaders recorded attendance for intervention groups, reporting 89.8% attendance in child session and 80% attendance in parental sessions [33].

Both the control and intervention schools were given a half-day seminar focusing on increasing knowledge about internalizing symptoms in children and how schools can support these children. Control schools used the existing structure for identifying and helping children with internalizing symptoms, e.g. treatment as usual (TAU).

For a complete description of the study protocol, see Patras et al. [29].

Comparisons between completers (n = 705) (defined as having data on at least two time points) and drop-outs (n = 90) showed no significant differences on symptoms measurements (MASC-C: *M*_completers_ = 61.76 [SD 14.07] versus *M*_*dropouts*_ = 63.64 [SD 13.48], *p* = .213. SMFQ-C: *M*_completers_ = 9.88 [SD 5.06] versus *M*_*dropouts*_ = 9.87 [SD 4.87], *p* = .992). (See Additional file 1: Table S1 for results from attrition analyses).

### Participants

A total of 1686 children participated in screening for anxious and depressive symptoms. Based on screening results, 873 children were invited to participate. Seven children were excluded due to exclusion criteria (e.g., mental retardation, autism, or being potentially unable to benefit from a group intervention), and 71 were excluded due to lack of resources (group leaders). Finally, 795 children were included in the study, and of these, 635 children (79.9%) had a parent participating in at least one of the measurement points (83.4% of the parents were mothers).

An income equal to or above the median income in Norway [34] was reported by 82.4% of the sample. Education at the college or university level was reported by 67.0% of parents. By comparison, a corresponding education level are reported by 32.9% of the general Norwegian population [35]. In our sample, 71.3% of the children lived with both their parents. Norway was reported as the place of birth for 95.3% of the children, for 88.8% of the mothers and for 89.2% of the fathers.

### The Intervention

The indicated EMOTION intervention is CBT based, and it focuses on teaching children adaptive symptom management skills. In the first half of the intervention, the emphasis is on psychoeducation and learning new coping skills. The second half is reserved for practicing the skills and learning to approach avoided situations through behavioral experiments [27]. Examples of strategies that target depressive symptoms include using coping strategies to improve mood, emotion regulation and behavioral activation, while strategies that targeted anxious symptoms include building a fear hierarchy and undergoing graded exposure. The parallel parent groups covered subjects that corresponded to those of the child groups, with the intention of increasing parental support for children and providing a general focus on positive parent strategies [36]. A pilot study reported positive results for the feasibility of the EMOTION intervention [37]. Table 1 displays the curriculum components the EMOTION program.

**Table 1:** EMOTION curriculum session by session

Child group		Parental group	
Session	Curriculum	Session	Curriculum
1	Introduction	1	Introduction
2	Recognizing emotions, coping and goal setting	2	Positive parenting
5–9	Problem solving	3	Positive reinforcement and psychoeducation
11	Exposure, cognitive restructuring	4	Exposure and behavioral activation
12–17	Exposure, positive self-schema, cognitive restructuring	5	Problem solving, exposure, behavioral activation
18–20	Integration of skills, exposure and Closure	6	Exposure, behavioral activation, cognitive restructuring
		7	Cognitions, and closure

Martinsen et al. [27]

### Measures

The Multidimensional Anxiety Scale for Children (MASC) [38] child and parent versions were used to assess anxious symptoms. The MASC includes 39 items that cover anxious symptoms over the past 2 weeks. Cronbach’s alphas were acceptable for both the child (α = .84 at pre, .91 at post, and .91 at follow-up) and parent (α = .72 at pre, .81 at post, and .92 at follow-up) versions in the present sample.

The Mood and Feelings Questionnaire-short form (SMFQ) [39] child and parent versions were used to assess depressive symptoms. The SMFQ includes 13 items that cover depressive symptoms over the past 2 weeks. In addition, we added one question about suicidal ideation from the MFQ long version [40]. Cronbach’s alphas were acceptable for both the child (α = .81 at pre, .87 at post, and .88 at follow-up) and parent (α = .88 at pre, .87 at post, and .92 at follow-up) versions in the present sample.

### Statistics

The data were analyzed using linear mixed models (LMM), with the MASC and SMFQ symptom measures for child and parent reports as dependent variables. In all mixed models, child was included as a random effect and time as a categorical fixed-effects variable using the three timepoints (pre, post, and 12 months after intervention), the interaction between intervention and time, child age and gender as covariates. Intention to treat analyses (ITT) was used [41].

LMM has the advantage of being unbiased under the missing at random assumption, while a complete case analysis would have been unbiased only under the stricter missing completely at random assumption. The LMM included data from all participants who had data from at least one timepoint in the analysis. All mixed models were repeated with the child nested within school and school as a second random effect. The results were essentially the same (see Additional file 2: Table S2 for results).

Schools were the unit of randomization, and due to feasibility considerations, randomization was only performed in the first wave of data collection; hence, each school was kept as either an intervention or a control school throughout the data collection period. We compared the intervention and control groups at baseline in terms of the child and parent versions of the MASC and SMFQ, child age, child gender and sociodemographic factors (*t* test for scale variables and Pearson’s Chi squared test for dichotomous variables). Completers (those who had data for at least one follow-up point) and dropouts were compared using Student’s *t*-test.

*P*-values < .05 were considered statistically significant, and 95% confidence intervals (CIs) are reported where relevant. The statistical analyses were performed using SPSS 24.

## Results

The baseline characteristics of the intervention and control groups are presented in Table 2. There were no statistically significant intergroup differences regarding child or parent gender, parental education or income. A significant age difference was found at baseline: the children in the intervention group were older than those in the control group (*M*_intervention_ = 10.20 [SD .95] versus *M*_control_ = 10.01 [SD .86], *p* = .015). However, this small age difference was not considered clinically meaningful. The intervention group scored higher for both anxious and depressive symptoms compared to the control group at baseline across all respondents (see Table 2). The differences between the intervention and control groups regarding symptom measurement are although significant, relatively small. To what degree this difference represents a clinical meaningful difference should be investigated in future studies. So far, cautions should be made upon interpretation of the results.

**Table 2:** Descriptive statistics at baseline split by group

	Intervention group (*N* 358)	Control group (*N* 437)	*p*-value
Child age	10.20 (.95)	10.01 (.86)	.015*
Child female	221 (61.7)	240 (54.9)	.053
Parent mother	222 (82.20)	257 (84.50)	.463
Education parent	3.88 (1.02)	3.88 (1.05)	.933
Family income	4.66 (1.23)	4.67 (1.26)	.974
MASC-C	64.70 (13.42)	62.39 (13.58)	.017*
MASC-P	45.90 (15.42)	40.63 (14.68)	<.001***
SMFQ-C	10.32 (5.21)	9.51 (4.58)	.019*
SMFQ-P	6.66 (5.06)	4.63 (4.35)	<.001***

Mean (*SD*) or *n* (%). (*N* = 795)

*MASC-C* The Multidimensional Anxiety Scale for Children-child report, *MASC-P* The Multidimensional Anxiety Scale for Children-parent report, *SMFQ-C* The Mood and Feelings Questionnaire-short form, child report, *SMFQ-P* The Mood and Feelings Questionnaire-short form, parent report

* p ≤ .05. ** p ≤ .01. *** p ≤ .001

Table 3 displays the results of the mixed model analyses. Comparing the baseline results to those at the 12-month follow-up, for child self-reported anxious symptoms, the intervention group changed more than the control group: difference 4.56, CI 1.83 to 7.29, *p *= .001. For child self-reported depressive symptoms, there was no statistically significant difference in symptom change between the intervention and control group: difference .69, CI − .22 to 1.60, *p *= .139.

**Table 3:** Results from mixed model analyses with MASC child and parent version, and SMFQ child and parent version as dependent variables

Time	Measurement	Intervention group		Control group		Difference (interaction between group and time)		*p*-value
		*N*	Mean (*SE*)	*N*	Mean (*SE*)	Estimate (95% CI)		
						Baseline to 12 months follow-up	Post-intervention to 12 months follow-up	
Baseline	MASC-C	358	63.90 (.94)	437	62.99 (.88)			
	MASC-P	268	45.57 (.96)	300	40.52 (.89)			
	SMFQ-C	358	10.41 (.32)	437	9.45 (.30)			
	SMFQ-P	267	6.69 (.28)	298	4.64 (.26)			
Post intervention	MASC-C	266	51.14 (1.00)	428	56.40 (.88)		2.63 (− .14 to 5.39)	.062
	MASC-P	194	43.50 (1.04)	230	38.87 (.98)		− 2.08 (− 4.49 to .34)	.092
	SMFQ-C	265	7.86 (.34)	428	7.65 (.30)		.07 (− .85 to .99)	.879
	SMFQ-P	193	5.06 (.31)	227	4.32 (.29)		− .23 (− 1.00 to .54)	.564
12 months follow-up	MASC-C	269	49.13 (.99)	406	51.76 (.90)	4.56 (1.83 to 7.29)		.001***
	MASC-P	193	41.57 (1.05)	239	39.03 (.98)	2.50 (.26 to 4.74)		.029*
	SMFQ-C	269	6.75 (.33)	406	6.48 (.30)	.69 (− .22 to 1.60)		.139
	SMFQ_P	188	4.43 (.31)	235	3.92 (.29)	1.55 (.83 to 2.26)		< .001***

Child as random effect, and time as a categorical fixed-effects variable using three time-points (pre, post, and 12 months after intervention), interaction between intervention and time, child age and gender as covariates

*MASC-C* The Multidimensional Anxiety Scale for Children-child report, *MASC-P* The Multidimensional Anxiety Scale for Children-parent report, *SMFQ-C* The Mood and Feelings Questionnaire-short form child report, *SMFQ-P* The Mood and Feelings Questionnaire-short form, parent report

* p ≤ .05. ** p ≤ .01. *** p ≤ .001

For parent reported child anxious symptom, the intervention group had changed more than the control group at 12 months after the intervention: difference 2.50, CI .26 to 4.74, *p *= .029. For parent reported child depressive symptom, the intervention group changed more than the control group at 12 months after the intervention: difference 1.55, CI .83 to 2.26, *p *≤ .001. There were no statistically significant differences between the intervention and control groups regarding the change over time from postintervention to the 12-month follow-up. This was true across all reporters, see Table 3.

**Fig. 2:**
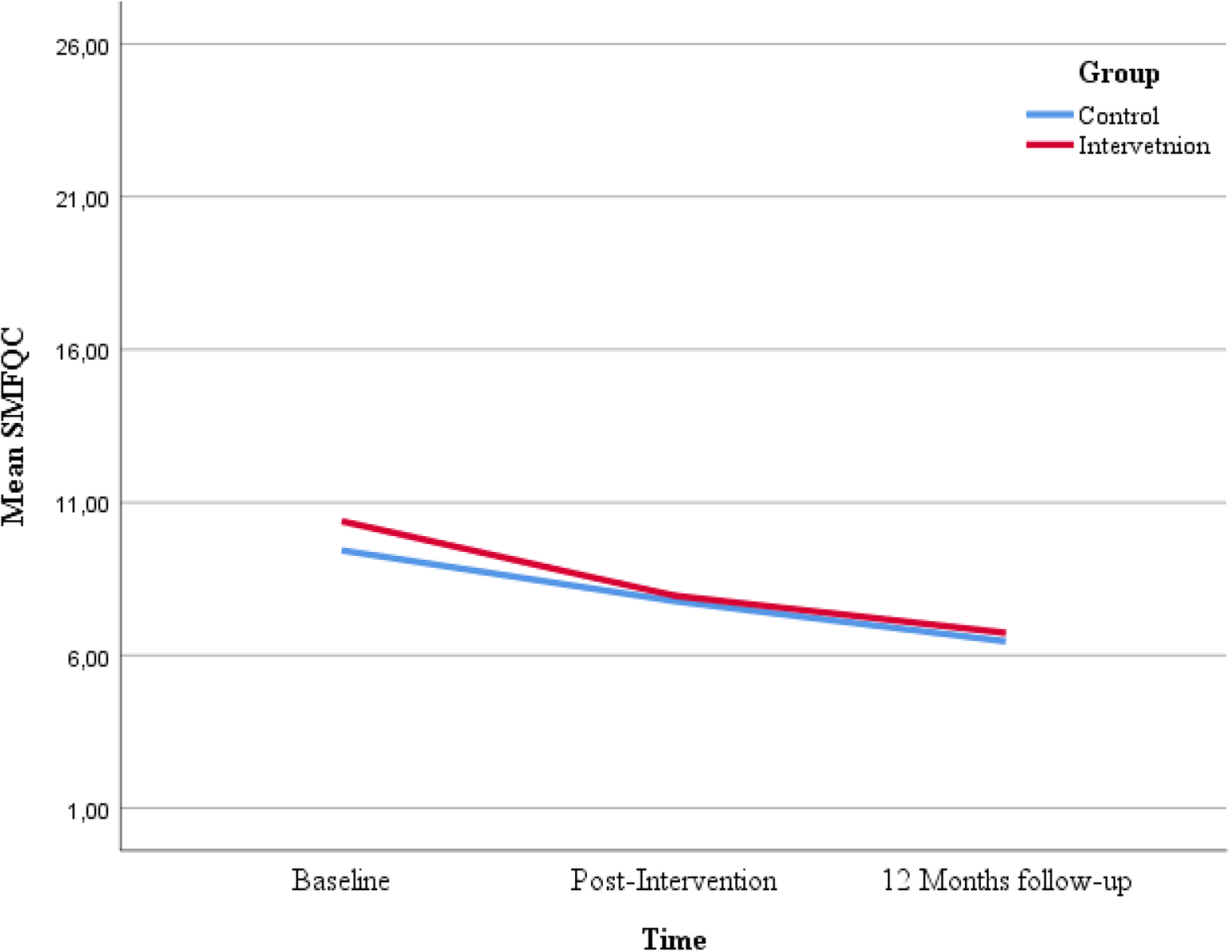
Comparison between groups, depressive symptoms, child report. *SMFQ-C* Mood and Feelings Questionnaire-short form

**Fig. 3:**
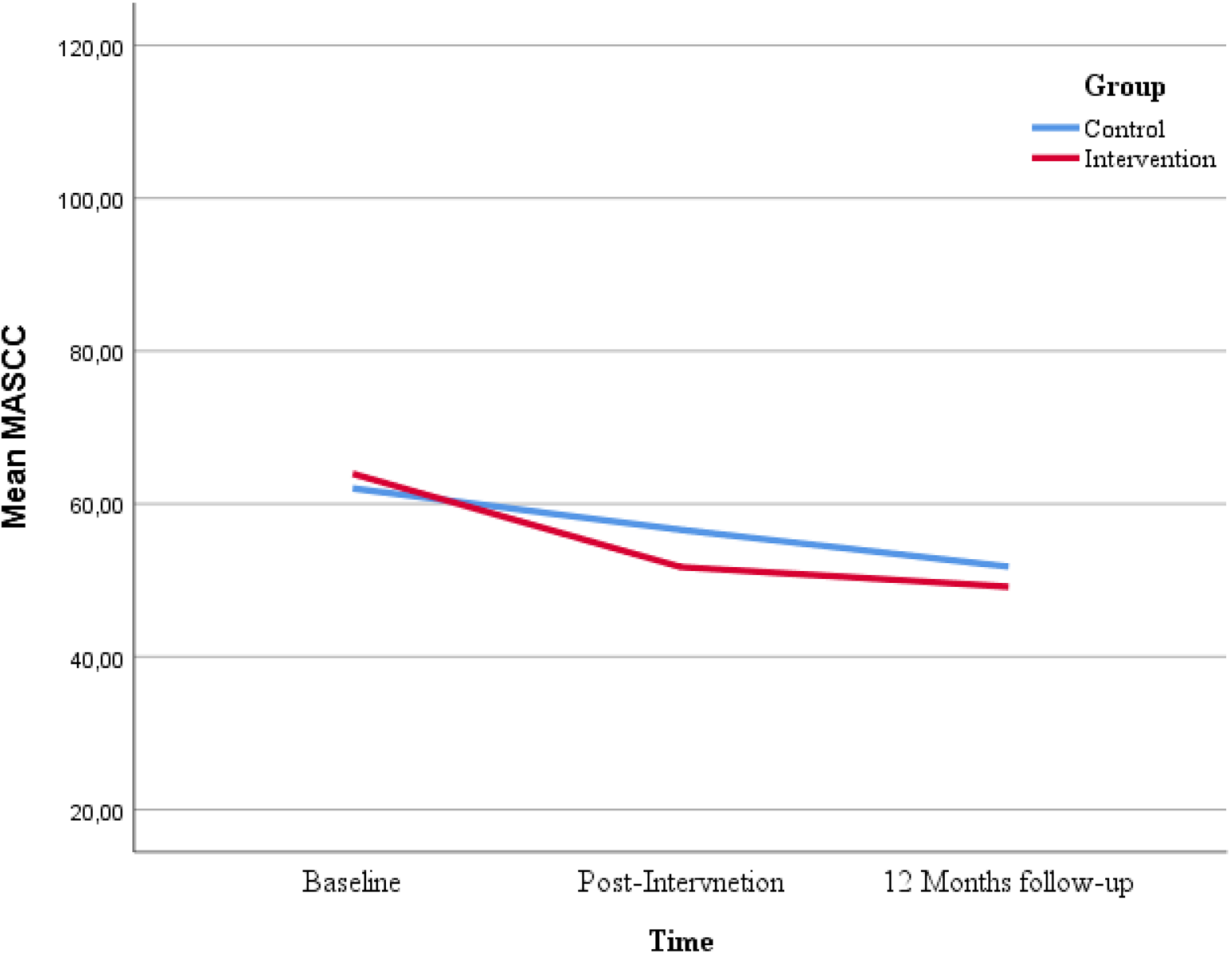
Comparison between groups, anxious symptoms, child report. *MASC* Multidimensional Anxiety Scale for Children

**Fig. 4:**
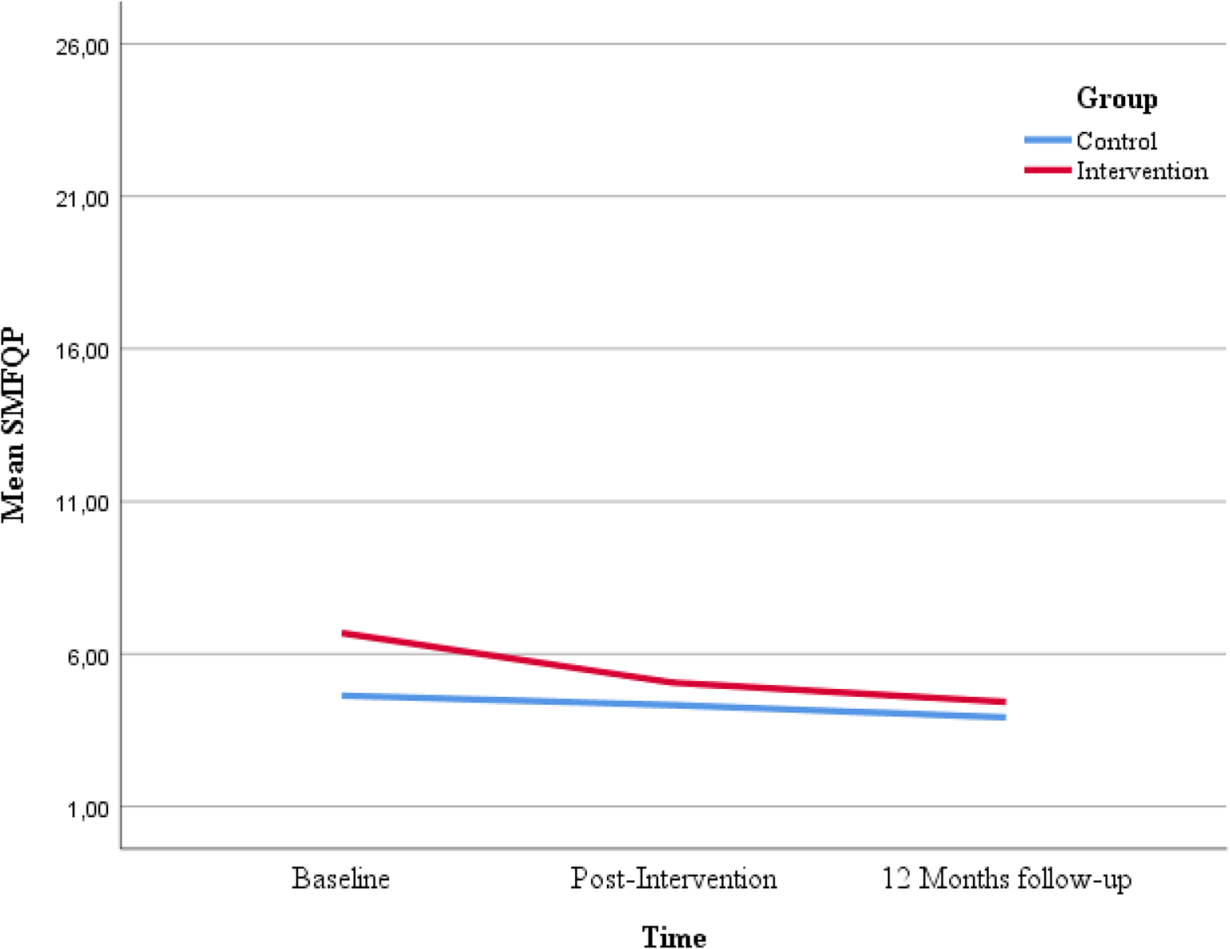
Comparison between groups, depressive symptoms, parental report. *SMFQ* Mood and Feelings Questionnaire-short form

**Fig. 5:**
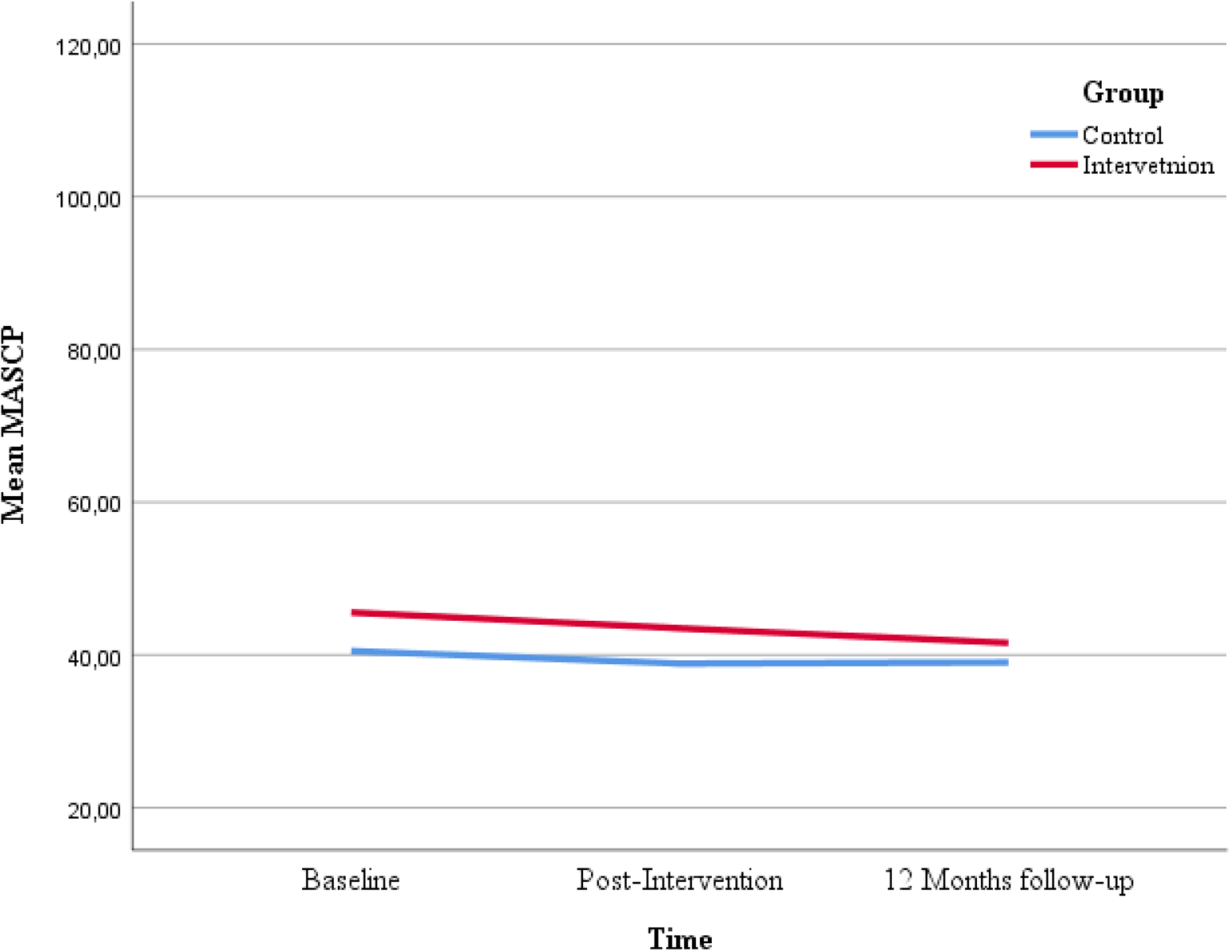
Comparison between groups, anxious symptoms, parental report. *MASC* Multidimensional Anxiety Scale for Children

Figures 2, 3, 4 and 5 displays the development of the intervention and control groups over time, split by symptoms and reporter. Insert Fig. 2, 3, 4 and 5.

## Discussion

For both child and parent reports of the children’s anxious symptoms, the symptom reduction in the intervention group was greater than in the control group 1 year after the intervention, indicating a small but significant effect of the EMOTION program. The effect of the EMOTION intervention persisted from baseline to the 12-month follow-up but did not continue to increase between the postintervention assessment and the 12-month follow-up.

The results of the children’s reports are consistent with the previously published postintervention effects of EMOTION [28], suggesting that the intervention has potential for effective prevention of anxious symptoms from both a short- and a long-term perspective. The mean score on MASC-C was reduced by 14 points from baseline to follow-up for the intervention group (see Table 3), a reduction approximately corresponding to one SD, which may be considered clinically meaningful for the child. Nevertheless, the mean MASC-C score in the control group was reduced by 11 points from baseline to follow-up, a change that may be large enough to represent a clinical meaningful difference for the child. The difference between the groups, although statistically significant, was only three points. It should be considered that school staff in control schools, as well as intervention schools, were given a half-day seminar to raise awareness about internalizing symptoms in children, and how to best support these children. Possibly, the seminar as well as the awareness raised by participating parents and children answering questions regarding internalizing symptoms, contributed to the symptom reduction in the control group.

The postintervention results of EMOTION indicated that there was no reduction in the children’s anxious symptoms as assessed by the parents [28]. The positive effect found after 12-months suggests a delayed effect of the EMOTION intervention on anxiety symptoms as reported by parents. The positive findings regarding follow-up reductions in anxiety symptoms across informants strengthen the results and indicates that the EMOTION intervention have the potential to create lasting improvements in children’s strategies for managing anxiety.

The small effect of the EMOTION program are in line with those of similar prevention programs, such as the FRIENDS for Life program [25] and a review focusing exclusively on school-based prevention programs for anxiety and depression [4]. However, the results of this study contradict reviews that report no effects for anxiety prevention at a 12-month follow-up [3, 9]. Recent reviews have especially called for more studies evaluating the follow-up effects of anxiety prevention, as the existing results are uncertain, primarily due to the small number of published studies [3, 4].

Furthermore, treatment interventions targeting children with diagnosed anxiety disorders show promising long-term results [42, 43]. The extent of the EMOTION intervention resembles treatment interventions, and it is possible that this sort of extensiveness is necessary for the successful prevention of anxiety symptoms.

For depressive symptoms, the parents reported a greater reduction in child symptoms in the intervention group compared to the control group at the 12-month follow-up, indicating a small but significant effect of the EMOTION program. The child reports did not support these findings, however, as no effects were found on depressive symptoms as assessed by the children. This finding is only partly in line with the pre- to postintervention results for the EMOTION program [28], where both child self-report and parental report of child symptoms showed an effect on depressive symptoms. The parent-reported results correspond with those of previous studies showing a small effect for depression in both the short- and long-term [3, 25]. Dissimilar results from different informants are common [44], and variations in effect across informants are also found in similar trials. For example, Kösters et al. [25] found positive effects using children’s self-reports on both anxiety and depression 12 months after the intervention, but teachers’ reports showed no effects, and peer reports indicated an increase in symptoms among those following the intervention. In support of the clinical importance of parent reported reduction in child depressive symptoms, others have found parental reports to be the best predictor of mood disorders in a group of children under the age of twelve [45]. The parents in the intervention group reported a mean reduction of three points on SMFQ from baseline to follow-up. Parental report of child symptoms was below cut of at baseline (as child reported symptoms was the inclusion criteria). Given the low symptom report at baseline a small reduction is expected as there is not much symptoms to reduce [46]. Further, in a longitudinal study of girls only from the age of 8 to 11 years old, reporting subclinical symptoms of depression, the risk of later developing a depressive disorder doubled for each reported symptom [47]. These results support the clinical importance of even small reduction of depressive symptoms. Nevertheless, as no effect was reported by children themselves, improvements should be made to the program in order to increase effect of the EMOTION intervention.

The uncertain results regarding depressive symptoms in the present study suggest that there is room for improvement in the EMOTION intervention. Research has suggested that effective regulation of dysphoric mood is important for recovery from juvenile depression [48]. However, the EMOTION intervention has been found to be effective for improving children’s general emotion regulation capacity [49], indicating other important factors relevant to the long-term prevention of depression should be improved. For example, improving group leader’s ability to set specific goals relevant for depressed children, working on maladaptive cognitive thinking and strengthen adaptive mood-repair [50] appears to be an area of improvement. Further improvement could possibly be to include booster sessions in the intervention [10]. The results regarding booster sessions are mixed, however [51, 52]. Booster sessions are characterized by considerable heterogeneity (e.g., in terms of the number of sessions and the content of sessions), which may contribute to the mixed findings. Possibly booster sessions emphasizing use of both mood-repairs and behavioral activation as a mean to regulate dysphoric mood would contribute to improvement in child-reported depressive results. However, this remains to be investigated.

The EMOTION intervention provides seven parent sessions, four of which include the children and three to which only the parents are invited. The parents in our sample were taught both how to recognize their child’s symptoms and how to support their child in implementing the skills and strategies acquired in the EMOTION program. A substantial amount of evidence supports the importance of parent-related factors, such as parental mental health and parenting practices, to children’s risk of internalizing symptoms [53]. Nevertheless, it is still equivocal whether parental involvement in interventions increases effectiveness [53, 54]. Possibly involving parents enhances the effectiveness of interventions for younger children [53, 54], and a recent meta-analysis found that parental involvement in CBT treatment targeting anxiety was associated with increased effects both postintervention and at follow-up [51]. The parental participation in the EMOTION intervention might have contributed to the finding that the parents in the intervention group reported greater symptom reduction than parents in the control group.

Results from the present study indicate that it is possible to prevent both anxious and depressive symptoms in one transdiagnostic intervention with lasting effects. Further, when conducting research on prevention interventions even a small effect is considered beneficial at a population level, as it may reduce the onset of disorders [4, 55]. This is in line with the results from Kösters et al. [25] and elaborates the findings of Martinsen et al. [28]. Transdiagnostic programs have the advantages of targeting a larger group of children, simplifying implementation, and potentially reducing costs [15, 16, 53]. In the present sample, variation among schools was insignificant. This, we expect is a result of the Norwegian school system, where the great majority of children go to public schools [56] and where economic inequality, both within and among schools, is relatively low. To improve the impact of preventive interventions for children, studies focusing on moderators and mediators of effects are needed to identify essential components of the EMOTION intervention and similar interventions. Furthermore, the use of new technology (e.g., Virtual Reality and web-based sessions) could be investigated to make the intervention more feasible within municipal service settings, to investigate the importance of the intensity of the intervention and potentially strengthen the effects.

### Strengths and limitations

The present study had a large sample size and high response rate at follow-up. The study was performed in naturalistic settings and had few exclusion criteria. At baseline, there were statistically significant differences between the intervention and control groups on both anxiety and depression measures across all respondents. Therefore, we used a mixed model for our analyses to adjust for these differences. In addition, the mixed model has the advantage of including data from all participants who had data on at least two time-points in the analysis, thereby minimizing the amount of missing data in the analysis [57].

The sample seems to be skewed toward parents with more education and average or above-average income levels. Although socioeconomic differences in Norway are relatively low, we cannot rule out the possibility that our sample is only representative of those children in higher socioeconomic classes. Low socioeconomic status is associated with an increased risk of psychopathology in children [58]. To ensure generalization to this high-risk group, future research should strive to include these families. Demographic data were reported by parents, and approximately 22% of the included children had no parents participating in the study, or demographic parental data was missing. Consequently, we have no demographic information about these children. The missing parental data may have contributed to the skewness in the sample. For future reference it would be desirable to collect some demographic information directly from children or use register-based data to obtain such information. In the Coping Kids study schools were matched on demographic factors prior to randomization, but no additional steps were taken to ensure inclusion of children from diverse socioeconomic backgrounds.

Inclusion in the present study was based on child self-report only. Although the pilot study of EMOTION suggested this to be sufficient [37], recruitment from multiple informants are often recommended [59]. Possibly additional inclusion of children referred by teachers, school health nurses, or parents could contribute to a more diverse socioeconomical status of the sample.

To evaluate a true long-term prevention effect, it will be necessary to have a longer follow-up period than 12 months [43].

Drop-out rates after the intervention started was low (n = 22 in the intervention group, n = 16 in control). However, in the intervention group 67 children dropped out pre intervention, compared to only 7 in the control condition (see Fig. 1, Consort statement). Reasons for drop-out was mainly parental time-constraint and parents not viewing the child’s problem as severe. Possibly the extensiveness of the EMOTION intervention, as well as inclusion based on child self-reported symptoms alone, might have contributed to the high dropout rates preintervention in the intervention group.

In the present study 71 children were excluded due to lack of resources (see Fig. 1), as there were not enough group leaders to run child group for all eligible children. For future reference, more attention should be paid to recruitment of enough group leaders to avoid having to exclude children due to lack of group leaders. Rasmussen et al. [13] found that group leaders reported time, resources and general support from leaders, as barriers to implementation of the Emotion program. Possibly more attention paid to freeing resources for the group leaders could also ease the recruitment of group leaders.

Time constraint seems to be important to consider when implementing a large-scale prevention intervention. In the Coping Kids study time constraint was a reason given by parents for drop-out and a barrier to implementation as reported by group leaders [13]. After completing the Coping Kids study the EMOTION manual underwent revision, and the program was made more flexible where group leaders may reduce the intervention from 20 to 16 child sessions [60]. We do however not yet know if this improves the challenges regarding time constraints. A more comprehensive discussion of facilitators and barriers to implementation in the Coping Kids study are presented by Rasmussen and colleagues [13].

## Conclusion

The transdiagnostic EMOTION program has shown potential for long-term reduction of symptoms of anxiety in school-aged children. This could have important implications for reducing potential suffering for children and families and for reducing the possible costs to society for treating disorders later in life. However, results regarding depressive symptoms should be considered preliminary as only parental report indicated effect.

## Supplementary information

**Additional file 1: Table S1.** Attrition analyses: Comparisons between Completers and Drop-outs split by symptom measurements.

**Additional file 2: Table S2.** Results from mixed model analyses with MASC child and parent version, and SMFQ child and parent version as dependent variables. Child and school as random effects, and time as a categorical fixed-effects variable using three time-points (pre, post, and 12 months after intervention), interaction between intervention and time, child age and gender as covariates.

## Data Availability

The datasets generated and/or analyzed during the current study are not publicly available due to privacy policy but are available from the corresponding author on reasonable request

## Abbreviations

CBT: Cognitive behavioral therapy
RCT: Randomized controlled study
MASC-C/P: The Multidimensional Anxiety Scale for Children—child/parent version
SMFQ-C/P: The Mood and Feelings Questionnaire—short form child/parent version
CAS-CBT: Competence and adherence for cognitive behavioral therapy
