# Supplementary material for: A 12-month follow-up of a transdiagnostic indicated prevention of internalizing symptoms in school-aged children: the results from the EMOTION study
Source: Child Adolesc Psychiatry Ment Health. 2020 Apr 22;14:15. doi: 10.1186/s13034-020-00322-w (PMC7178617; doi:10.1186/s13034-020-00322-w)
Supplement: Supplementary file 1 — Additional file 1: Table S1. Attrition analyses: Comparisons between Completers and Drop-outs split by symptom measurements. [file 13034_2020_322_MOESM1_ESM.docx]

| **Additional material 1: Table S1. Attrition analyses:** Comparisons between Completers and Drop-outs split by symptom measurements. | | | | | | | |
| --- | --- | --- | --- | --- | --- | --- | --- |
|  | Completes | | | Drop-outs | | | *p* |
|  | N | M | SD | N | M | SD |  |
| MASC-C | 705 | 61.76 | 14.07 | 90 | 63.64 | 13.48 | .213 |
| MFQ-C | 705 | 9.88 | 5.06 | 90 | 9.87 | 4.87 | .992 |
|  |  |  |  |  |  |  |  |
| MASC-F | 521 | 43.46 | 15.36 | 106 | 41.63 | 14.72 | .266 |
| MFQ-F | 514 | 5.68 | 4.82 | 109 | 5.22 | 4.77 | .375 |
|  |  |  |  |  |  |  |  |
| Note: Participation in the study only required child data at T1. Parents who failed to answer T1, but had children participating in the study, were requested to participate at T2 and T3. Both parents of the child were requested to participate. Due to this, there were 624 children participating at T1, but 627 parents participating in the study overall. | | | | | | | |
