# Supplementary material for: A 12-month follow-up of a transdiagnostic indicated prevention of internalizing symptoms in school-aged children: the results from the EMOTION study
Source: Child Adolesc Psychiatry Ment Health. 2020 Apr 22;14:15. doi: 10.1186/s13034-020-00322-w (PMC7178617; doi:10.1186/s13034-020-00322-w)
Supplement: Supplementary file 2 — Additional file 2: Table S2. Results from mixed model analyses with MASC child and parent version, and SMFQ child and parent version as dependent variables. Child and school as random effects, and time as a categorical fixed-effects variable using three time-points (pre, post, and 12 months after intervention), interaction between intervention and time, child age and gender as covariates. [file 13034_2020_322_MOESM2_ESM.docx]

| **Additional material 2: Tale S2.** Results from mixed model analyses with MASC child and parent version, and SMFQ child and parent version as dependent variables. Child and school as random effects, and time as a categorical fixed-effects variable using three time-points (pre, post, and 12 months after intervention), interaction between intervention and time, child age and gender as covariates. | | | | | | | | |
| --- | --- | --- | --- | --- | --- | --- | --- | --- |
| Time | Measurement | Intervention group | | Control group | | Difference (interaction between group and time) | |  |
|  |  | *N* | Mean (*SE*) | *N* | Mean (*SE*) | Estimate (95% CI) | | *p*-value |
|  |  |  |  |  |  | Baseline to 12 months follow-up | Post-intervention to 12 months follow-up |  |
| Baseline | MASC-C | 358 | 63.91 (1.02) | 437 | 61.98 (.99) |  |  |  |
|  | MASC-P | 268 | 45.34 (1.14) | 300 | 40.74 (1.08) |  |  |  |
|  | SMFQ-C | 358 | 10.35 (.39) | 437 | 9.43 (.37) |  |  |  |
|  | SMFQ-P | 267 | 6.67 (.32) | 298 | 4.70 (.30) |  |  |  |
| Post intervention | MASC-C | 266 | 51.15 (1.07) | 428 | 56.39 (.96) |  | -2.62 (-5.38 to .14) | .063 |
|  | MASC-P | 194 | 43.27 (1.21) | 230 | 39.08 (1.15) |  | 2.08 (-.33 to 4.49) | .090 |
|  | SMFQ-C | 265 | 7.80 (.40) | 428 | 7.64 (.37) |  | -0.07 (-.99 to .85) | .888 |
|  | SMFQ-P | 193 | 5.04 (.34) | 227 | 4.38 (.33) |  | 0.23 (-.54 to 1.00) | .556 |
| 12 months follow-up | MASC-C | 269 | 49.13 (1.07) | 406 | 51.75 (.98) | 4.56 (1.83 to 7.29) |  | .001*** |
|  | MASC-P | 193 | 41.34 (1.22) | 239 | 39.23 (1.15) | 2.50 (.25 to 4.73) |  | .029* |
|  | SMFQ-C | 269 | 6.69 (.40) | 406 | 6.46 (.37) | 0.69 (-.22 to 1.59) |  | .138 |
|  | SMFQ_P | 188 | 4.41 (.35) | 235 | 3.98 (.33) | 1.55 (.83 to 2.26) |  | <.001*** |
| *p= <0.05. **p= <0.01. ***p= <0.001. | | | | | | | | |
| Note: MASC-C = The Multidimensional Anxiety Scale for Children – child report. MASC-P = The Multidimensional Anxiety Scale for Children – parent report. SMFQ-C = The Mood and Feelings Questionnaire – short form, child report. SMFQ-P = The Mood and Feelings Questionnaire – short form, parent report. | | | | | | | | |
